# Symptoms Associated with Vestibular Impairment in Veterans with Posttraumatic Stress Disorder

**DOI:** 10.1371/journal.pone.0168803

**Published:** 2016-12-29

**Authors:** Yaa O. Haber, Helena K. Chandler, Jorge M. Serrador

**Affiliations:** 1 Department of Pharmacology, Physiology and Neuroscience, Rutgers Biomedical Health Sciences, Newark, New Jersey, United States of America; 2 War Related Illness and Injury Study Center, Veterans Affairs New Jersey Healthcare System, East Orange, New Jersey, United States of America; 3 Cardiovascular Electronics, National University of Ireland Galway, Galway, Ireland; Central Institute of Mental Health, GERMANY

## Abstract

Posttraumatic stress disorder (PTSD) is a chronic and disabling, anxiety disorder resulting from exposure to life threatening events such as a serious accident, abuse or combat (DSM IV definition). Among veterans with PTSD, a common complaint is dizziness, disorientation and/or postural imbalance in environments such as grocery stores and shopping malls. The etiology of these symptoms in PTSD is poorly understood and some attribute them to anxiety or traumatic brain injury. There is a possibility that an impaired vestibular system may contribute to these symptoms since, symptoms of an impaired vestibular system include dizziness, disorientation and postural imbalance. To our knowledge, this is the first report to describe the nature of vestibular related symptoms in veterans with and without PTSD. We measured PTSD symptoms using the Posttraumatic Stress Disorder Checklist (PCL-C) and compared it to responses on vestibular function scales including the Dizziness Handicap Inventory (DHI), the Vertigo Symptom Scale Short Form (VSS-SF), the Chambless Mobility Inventory (CMI), and the Neurobehavioral Scale Inventory (NSI) in order to identify vestibular-related symptoms. Our findings indicate that veterans with worse PTSD symptoms report increased vestibular related symptoms. Additionally veterans with PTSD reported 3 times more dizziness related handicap than veterans without PTSD. Veterans with increased avoidance reported more vertigo and dizziness related handicap than those with PTSD and reduced avoidance. We describe possible contributing factors to increased reports of vestibular symptoms in PTSD, namely, anxiety, a vestibular component as well as an interactive effect of anxiety and vestibular impairment. We also present some preliminary analyses regarding the contribution of TBI. This data suggests possible evidence for vestibular symptom reporting in veterans with PTSD, which may be explained by possible underlying vestibular impairment, worthy of further exploration.

## Introduction

Posttraumatic stress disorder (PTSD) is defined as an anxiety disorder resulting from an event that evokes extreme fear, helplessness, or horror such as an accident, physical or emotional abuse or combat. The Diagnostic and Statistical Manual of Mental Disorders (DSM-IV) identifies three clusters of symptoms that comprise PTSD: intrusive thoughts and re-experiencing of the trauma in dreams and nightmares, avoidance of circumstances of the trauma and a persistent hyperarousal state often including hypervigilance and an exaggerated startle response. Although not part of the classification criteria, anecdotes of patients with PTSD in our clinic have described symptoms such as dizziness, disorientation, and or extreme discomfort in environments such as grocery stores, malls and stadiums as well as subsequent avoidance of these types of situations. These symptoms are typically viewed as manifestations of the anxiety disorder but they are also characteristic of symptoms experienced by patients with vestibular dysfunction such as visual vertigo or space and motion discomfort [1]. The current study, therefore, sought to quantify the extent to which veterans with PTSD report symptoms and behavior consistent with impaired vestibular function as well as to assess its impact on functioning.

The vestibular apparatus is located in the inner ear and its proper function is critical for postural control, maintaining equilibrium and spatial awareness. Neuroanatomical mapping by Balaban et al, Azzena et al, Liu et al has shown connections from the vestibular system to areas of the brain associated with anxiety such as the hypothalamus, the cerebellum and the amygdala [2–5]. Current literature on anxiety and vestibular function indicate that there may be a relationship. Furman et al investigated the vestibular ocular reflex of panic disorder patients and found increased gains and a reduced time constant which may be an indication of poor peripheral vestibular dysfunction [6]. In 2010, Teggi et al compared panic disorder patients to age matched controls and identified that panic disorder patients were at greater risk for abnormal posturography scores [7], demonstrating a problem with balance.

While extensive research has been performed on vestibular function and patients with panic disorder, there is currently a knowledge gap regarding reports of vestibular symptoms and PTSD. A number of studies have noted an association between PTSD and self-reported dizziness, including Baker et al [8]. They observed that veterans with PTSD had 16.2 times greater odds of reporting dizziness compared to those without PTSD (see also Macera et al, 2012; Hinton et al, 2013). However, these studies relied either on PTSD screening instruments (Macera et al, 2012) or single items rather than validated instruments to assess dizziness [8, 9].

This report seeks to use widely validated tools for assessing symptoms of PTSD and vestibular symptoms to determine if there is a relationship between self-reported PTSD and symptoms of an impaired vestibular system. We hypothesized that PTSD would be associated with increased reporting of vestibular symptoms, dizziness-related functional impairment, and avoidance of visually-challenging environments. Since avoidance symptoms are worse in vestibular patients, we anticipated that increased avoidance symptoms would be associated with increased vestibular symptoms. We expected that veterans with PTSD and increased avoidance symptoms would endorse more vestibular impairment symptoms and that they would cope with underlying vestibular dysfunction by avoiding visually challenging environments.

## Methods

All participants received written informed consent prior to participation. Informed consent was obtained using a paper consent form. Subjects were only enrolled if they reviewed and signed the form. This study was approved by the Human Studies Subcommittee (IRB) VA New Jersey Healthcare System under ID # MIRB 01140. This report is a subset of data from a larger prospective study seeking to characterize vestibular and autonomic function following military deployment-related blast exposure. The study was approved by the local VA NJ Health Care System Institutional Review Board in accordance with Helsinki standards. The study included Veterans age 18 and older from all conflict eras and excluded veterans with a recent history of use of vestibular suppressant such as benzodiazepines within the previous 30 days and or a history of drug or alcohol abuse within the previous 6 months of study participation. The current report focuses on the relationship between PTSD and vestibular symptoms.

### 2.1 Posttraumatic Stress Disorder Checklist (PCL-C)

The PCL-C is a 17 item self-report scale for PTSD based on Diagnostic Statistical Manual-IV (DSM-IV) criteria. PCL scores have a high internal consistency ranging from 0.94 to 0.97 [10, 11]. Additionally, the test retest reliability is also high ranging between 0.96 at 2–3 days and 0.88 at 1 week [11, 12]. We utilized a cutoff score of 37 based on the finding that scores ranging between 30-low 40’s have been found to be highly sensitive for capturing PTSD symptoms of veterans in primary care settings [13–15]. Thus subjects scoring above 37 were classified as “PTSD,” and all other subjects were considered “non PTSD.”

### 2.2 Neurobehavioral Symptom Inventory (NSI) Tool

This questionnaire is primarily used in the Veterans Health Administration as a component of a larger comprehensive traumatic brain injury evaluation. It is administered using a semi- structured interview format and provides a more in depth assessment of neurobehavioral symptoms ranging from dizziness to cognitive alertness. While it is primarily aimed at soldiers from Operation Enduring Freedom and Operation Iraqi Freedom, we used it for all study participants. It has high internal consistency with Cronbach alpha = 0.95 and high test reliability 0.88 to 0.93 [16]. Within this scale, we generated a vestibular subscale that included four items, i.e. feeling dizzy, loss of balance, poor coordination and sensitivity to noise. Our vestibular subscale had a Cronbach’s alpha = 0.75. This evaluation was added after participants were already enrolled, and thus we were only able to collect this data for 67 participants.

### 2.3 Dizziness Handicap Inventory (DHI)

This is a screening tool that aims to identify possible sources of handicap resulting from dizziness as a symptom. It is a 25 item self-report tool where subjects must respond with “always” (scored with a 4), “sometimes” (scored with a 2) or “no” (scored with a 0). There are three subscales within the scale which include physical, emotional and functional handicaps. The DHI has been reported to have high internal consistency ranging from Cronbach’s alpha 0.88 to 0.95 [17]. There is also high test retest reliability of the DHI at 0.90. This data collection tool was also added after enrollment was initiated thus only 59 of our participants had completed DHI forms.

### 2.4 Vertigo Symptom Scale Short form (VSS-SF)

This is a fifteen item self-report scale that is composed of two subscales. Eight items evaluate vertigo and balance and seven items evaluate autonomic anxiety. Scores can range between 0 and 28 with scores above 12 being defined as severe dizziness. Among patients with vestibular dysfunction, this test has been rated as having excellent test retest reliability at r = 0.94 for vertigo and 0.95 for the autonomic/anxiety symptom scale [18]. Internal consistency of the VSS has a Cronbach alpha range from 0.76 to 0.92 [18–20]. We collected the VSS-SF from 61 participants as the study was already ongoing.

### 2.5 Chambless Mobility Inventory (CMI)

This is a scale that aims to have participants self-report any anxiety or discomfort they may experience within specified locations and situations when alone or accompanied by a trusted individual. The internal consistency is 0.96 for avoidance when alone versus 0.95 when accompanied by a trusted individual. The construct validity ranges from 0.55 to 0.88 while the discriminant validity ranges from 0.28 to 0.29 [21, 22]. We collected 64 inventories from our participants.

### 2.6 Assessment of Traumatic Brain Injury (TBI)

In order to assess a history of TBI, we performed a Polytrauma Interview which is a semi structured interview for assessing lifetime TBI incidents. We classified TBI’s based on an inter-rater system where two trained individuals provided judgment of TBI status with a third rater providing their assessment if the first two raters were not in agreement. This evaluation was initiated after some participants were already enrolled, and thus we were only able to collect this data for 67 participants_._

### 2.7 Statistical Analysis

All data were analyzed using SPSS Statistical Software (IBM, Version 24). We utilized an independent samples student’s t-test to compare veteran responses to PCL, DVBIC and CMI. Additionally we utilized a linear regression Analysis of Variance (ANOVA) model to compare PCL score with the DHI, NSI and the VSS and a least squares differences post hoc analysis. For all findings, significant results were based on p value less than 0.05 and a two tailed analysis.

## Results

### 3.1 Study Participant Characteristics

At the time of this analysis 106 subjects were consented. Following consent, 17 individuals were excluded because they did not meet all of the inclusion criteria, leaving 89 enrolled. For PCL analysis, 2 individuals did not have enough time to complete the questionnaire and this left 87 PCL for analysis. Among the 87 completed PCL-C questionnaires, 37 veterans had a total score below the 37 cutoff on the PCL-C, indicating that they would not be classified as having PTSD. Meanwhile 50 of the subjects had a score above 37, meeting the requirement to be classified as having PTSD. Therefore, in our sample, 42.5% were considered to be negative for PTSD and 57.4% to be positive for PTSD. Of the 37 in the group without PTSD, the mean age was 46 years with a standard deviation of 14.2. Similarly of the 50 veterans in the group with PTSD the mean age was 43.1 with a standard deviation of 12.2. There were 32 males in the non PTSD group. In the PTSD group, there were 47 males. When asked about a medical history of falls 4 (10.8%) of the veterans in the non PTSD group endorsed falls as opposed to 9 (18%) in the group with PTSD. Similarly when asked about a history of dizziness, 3 (8%) of the non PTSD group versus 13 (26%) of the group with PTSD reported a history of dizziness (see Table 1).

**Table 1:** Study population characteristic information.

*Demographics*	Non PTSD (N = 37)	PTSD (N = 50)	Total
***Age***	46 ±14.1	43 ± 12.3	44.4 ± 13.1
***Males***	32 (87)	47 (94)	79 (91)
***History of Falls***	4 (11)	9 (18)	13 (15)
***History of Dizziness***	3 (8)	13(25)	16 (18)

Groups are based on veteran self-report on Posttraumatic Stress Disorder Checklist (PCL-C) where veterans scoring below 37 are classified as non PTSD and those with a score above 37 are classified as PTSD. Note *Percentages are in Parenthesis.

### 3.2 Vestibular Subscale of the Neurobehavioral Symptom Inventory (NSI)

**Fig 1:**
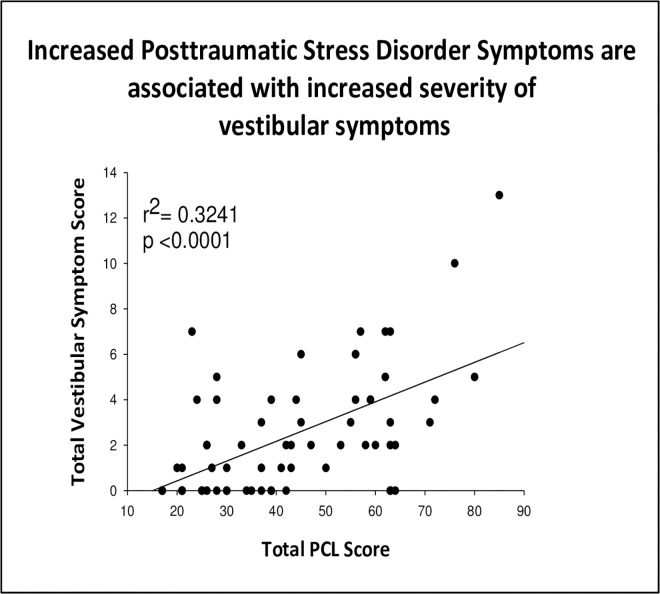
Regression analysis between total PCL score and total vestibular symptom subscale (VSS-V). Increased PTSD symptoms are associated with increased vestibular symptom endorsement within 30 days of study participation (r^2^ = 0.32, n = 66, p< 0.0001, two tailed).

When participants were asked about the extent to which they experienced neurological and behavioral symptoms within the previous 30 days of study participation we noticed that in all the categories, Veterans with PTSD reported an increased severity of symptoms. For the vestibular subscale related symptoms, such as *feeling dizzy*, *loss of balance*, *poor coordination and headaches*, a linear regression was conducted to compare the total score on the PCL with the total score for these 4 symptoms. Veterans with PTSD reported more symptoms than veterans without PTSD (p < .0001). Veterans with worse PTSD symptoms experienced a greater number of vestibular related symptoms within 30 days of study participation as illustrated in Fig 1.

### 3.3 Dizziness Handicap Inventory

**Fig 2:**
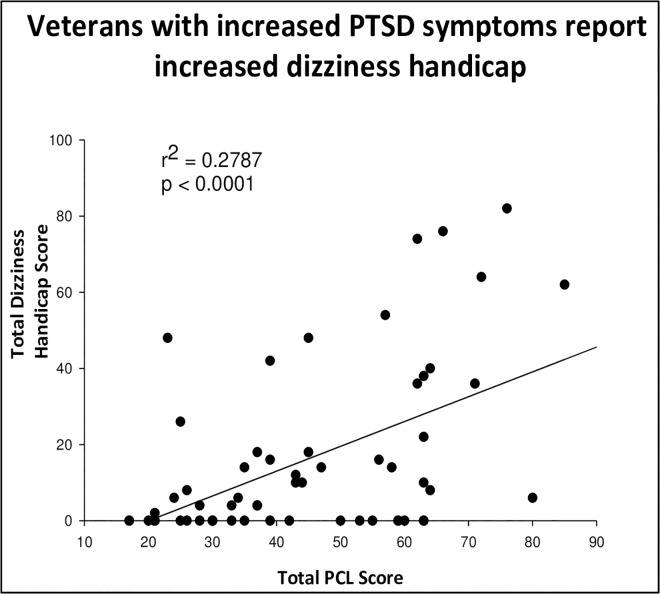
The correlation between total PCL score and total dizziness handicap score. A higher PCL score indicates more severe PTSD symptoms while a higher DHI score indicates a greater handicap due to dizziness symptoms. Total PCL score and DHI score were significantly correlated (r^2^ = 0.34, n = 62, p< 0.0001, two tailed).

We performed a linear regression model of the effect of PCL on Dizziness Handicap Inventory (DHI). DHI was significantly correlated to PCL scores, suggesting that Veterans with more severe PTSD symptoms also had more dizziness handicap as illustrated in Fig 2 (r^2^ = 0.28, p< 0.0001). Overall, veterans with PTSD reported increased dizziness symptoms compared to veterans without PTSD. Their dizziness symptoms were also associated with severe functional, physical and emotional handicap.

Looking more closely at specific symptoms, PTSD is predictive of increased dizziness severity (p<0.002, r^2^ = 0.16). While it was anticipated that veterans with PTSD would experience increased dizziness in supermarkets compared to veterans without PTSD, this was not statistically significant (mean PTSD group = 0.72±0.86, mean non-PTSD group = 0.23±1.20, p = .064). However, veterans with PTSD reported that quick head movements increased symptoms of dizziness to a greater extent than veterans without PTSD (mean PTSD group = 1.78±1.2, mean non-PTSD group = 0.62±1.7, p = 0.003).

In addition we performed a crosstabs analysis to quantify the distribution of handicap between the two groups. Within our sample of veterans, most of the participants experienced a mild handicap (77.6%). However among Veterans with PTSD, 12 people (34.3%) reported moderate or severe impairment whereas only one person (4.3%) reported moderate impairment in the non-PTSD group and none reported severe handicap. A chi square analysis of PTSD with DHI severity indicated that the relationship between PTSD status and dizziness handicap severity is significant (χ^2^ = 7.35, df = 2, N = 58 p = 0.025).

### 3.4 Vertigo Symptom Scale

**Fig 3:**
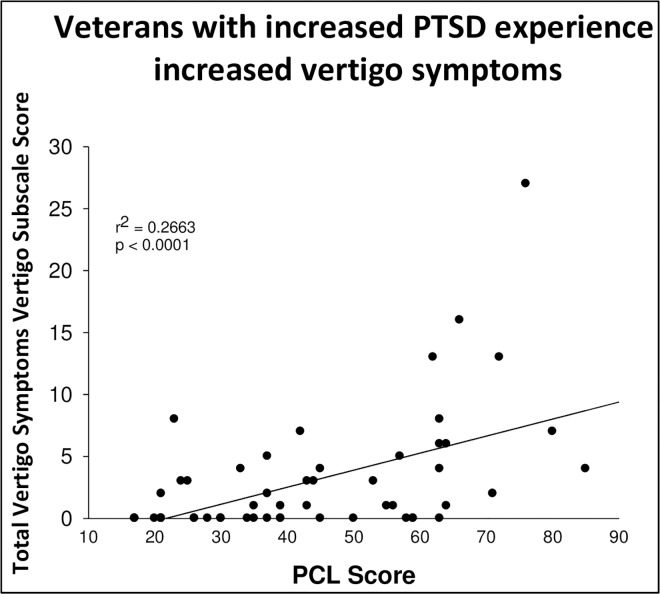
The correlation between PCL score and self-reported vertigo symptoms. As veterans scored higher on PCL indicating more PTSD symptoms, there was also a tendency towards higher scores on the vertigo self-report scale (r^2^ = 0.27, n = 55, p < 0.0001, two tailed).

Veteran responses to items about severity of symptoms to the vertigo symptom short form indicated that veterans with PTSD report more severe symptoms than veterans without PTSD. A correlation between total PCL score and total score on vertigo related items indicated that as PCL score increased, an indication of greater PTSD symptoms, so did vertigo symptom total score, an indication of vertigo symptom severity. This relationship is illustrated in Fig 3 (r^2^ = 0.27, p<0.0001). Veterans in the PTSD group endorsed more vertigo symptoms than veterans in the non PTSD group.

### 3.5 Chambless Mobility Inventory

**Fig 4:**
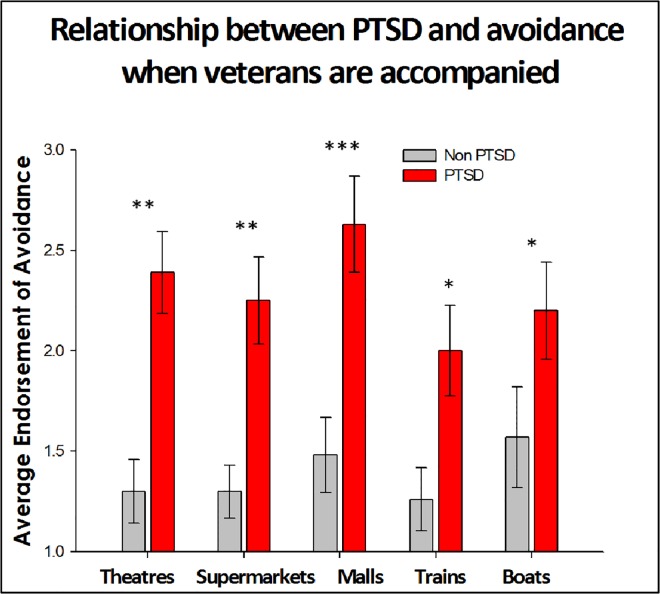
A comparison of veteran self-reports on Chambless Mobility Inventory when accompanied. Veterans with PTSD report increased discomfort and or avoidance of theatres, malls and supermarkets when accompanied by someone they trust. Note * denotes p< 0.05, ** denotes p <0.005 and ***denotes p < 0.0005

**Fig 5:**
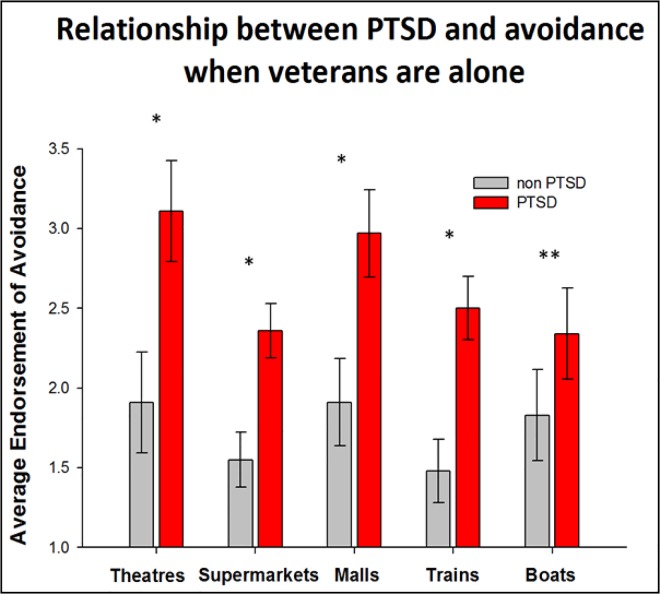
A comparison of veteran self-reports of avoidance when alone. Veterans report a greater discomfort or avoidance when alone in theatres, malls and supermarkets when they are by themselves. Note * denotes p< 0.05, ** denotes p <0.005.

Patients with vestibular dysfunction avoid certain environments because their symptoms become exacerbated by places such as theatres, malls and supermarkets. We utilized the Chambless Mobility Inventory to measure the extent to which Veterans with PTSD avoided theatres, malls, supermarkets and restaurants when they were alone. The data indicate that veterans with PTSD avoided these environments to a greater extent than veterans without PTSD. This trend was similar when veterans were accompanied. However for the item on restaurants, there were no significant differences between avoidance when veterans were alone compared to when accompanied. For items when accompanied, veterans with PTSD reported more avoidance symptoms in theatres p = 0.001 compared to veterans without PTSD. This was also true for supermarkets p = 0.001 and malls p < 0.0001, trains p = 0.009 and boats p = 0.007. When we compared veteran anxiety when alone, we found a similar response pattern such that veterans with PTSD avoided theatres p = 0.020, supermarkets p = 0.007, and malls, p = 0.004, trains p = 0.005 and boats p = 0.004 more than veterans without PTSD. The comparisons between the groups are illustrated in Figs 4 and 5.

Since Furman et al observed that patients with increased agoraphobia or avoidance symptoms experienced worse space and motion discomfort[1], we anticipated that high avoidance symptoms would be associated with worse vestibular function. Correlation analysis of avoidance and the total DHI and VSS scores indicated a high correlation between the mean avoidance severity score, with DHI (*R* = 0.55 p<0.0001) and VSSV (*R* = 0.77 p <0.0001). Mean severity score was assessed by averaging 7 avoidance items on the PCL-C.

### 3.6 TBI Status and Vestibular Symptoms

We used an inter-rater system to determine head injury status and performed an analysis of the incidence of a history of head injury between the PTSD and non PTSD group. We found that 46.4%of the non PTSD group had a history of head injury. Conversely, 72.5% of the PTSD group had a history of head injury. In order to determine if the high rates of head injury in the PTSD group contributed to the increased symptom reports, we performed a sub-analysis with 53 Veterans who had completed both the polytrauma interview and the VSS-V and 57 veterans who completed polytrauma and DHI. We performed a GLM univariate regression using both PTSD and TBI status as independent factors. From this analysis, we found that there was no significant effect of TBI on VSS-V, and there was no interaction with PTSD status. Similarly, when we performed the analysis on the DHI scores, we found no significant effect of TBI status and no interaction with PTSD status (see Table 2).

**Table 2:** Effect of comorbid head injury on total vertigo and dizziness handicap symptoms reported.

	*VSS-V*		*DHI*	
	TBI-	TBI+	TBI-	TBI+
**PTSD-**	1.0± 0.8^a^	0.6±0.4^a^	5.2±4.0^c^	5.6±2.7^c^
**PTSD+**	5.0±1.7^b^	4.2±1.3^b^	26.4±8.7^d^	22.7±4.8^d^

Veterans with PTSD report more vestibular symptoms irrespective of head injury status on both vertigo symptom scale and dizziness handicap. (VSS_V—vertigo symptom scale vestibular, DHI—dizziness handicap inventory). Values are mean score on the scale ± SEM. Veterans with PTSD had significanly higher VSS-V scores regardlesss of TBI status(b>a, p<0.05). Similarly they also had significantly higher DHI scores regardless of TBI status (d>c, p<0.05). There was no statistically signifcant difference in values between TBI groups regardless of PTSD status in both scales.

## Discussion

The sample population examined here were representative of the national veteran population. This sample was mostly male, from all conflict eras and the mean age was 44 years old. Regarding head injury,70% of the veterans were comorbid for a history of head injury which was higher than rates others have found, ~ 39% [23]. This may be partly due to the fact that this study was targeted at individuals with a history of head injury.

**Fig 6:**
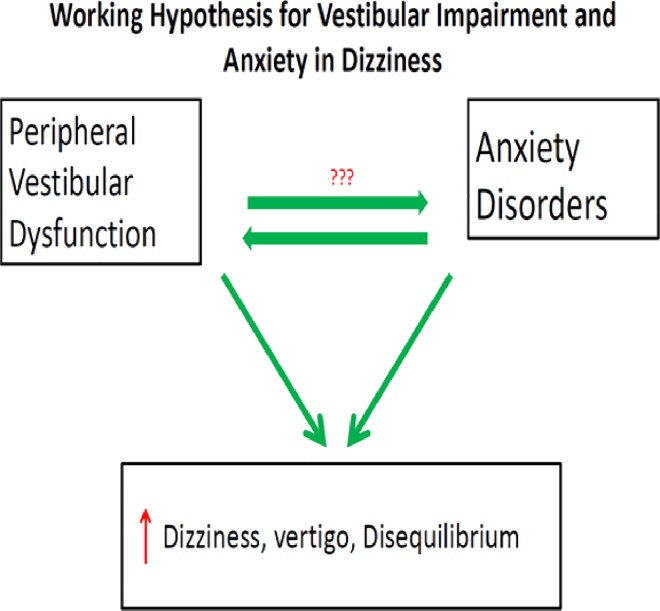
A working hypothesis of the relationship between vestibular function and anxiety. Anxiety and peripheral vestibular dysfunction have been associated with increased fall risk. Additionally both anxiety and peripheral vestibular dysfunction may interact and therefore lead to increased dizziness.

The major finding of this work is that in our sample, Veterans with PTSD reported greater incidence and severity of symptoms associated with vestibular impairment. Additionally, self-reported vestibular symptoms were associated with a greater level of dizziness related handicap. To our knowledge this is the first study of this type to provide data that symptoms of vestibular impairment are evident in PTSD using measures designed specifically to assess vestibular symptoms. Further, data from this study demonstrate that veterans with PTSD identify these symptoms as a source of disability. Our data provided evidence that symptom reporting within the two groups (PTSD and non PTSD) were not affected by head injury. Lastly, the PTSD group endorsed more vestibular symptoms on both the VSS and the DHI than the non PTSD group. There are several possible factors that may contribute to the symptoms of dizziness evidenced in the patients with PTSD. Currently we are exploring three possible sources that may explain these symptoms; anxiety, peripheral vestibular dysfunction and a combined effect of anxiety with peripheral vestibular dysfunction (see Fig 6).

### 4.1 Role of Anxiety in Vestibular Symptoms

Extensive data has shown that anxiety and dizziness are commonly associated [1, 2, 24, 25]. It is conceivable that anxiety levels of patients with PTSD are the main contributor to the symptoms of dizziness reported by the patients described in this study. Yardley et al found that anxiety and arousal are predictors of vertigo severity in a longitudinal study of patients with vertigo [18, 26, 27]. In that work, elevated anxiety was associated with self-reported dizziness related handicap [18]. Examining our study sample, we also observed that the veterans with PTSD report high levels of symptomatology on the vertigo symptom scale and on the dizziness handicap inventory.

To further explore the role of anxiety in the reported vestibular symptoms, we performed a post hoc analysis using a linear regression model with the predictors of vestibular related vertigo (VSS-V) and anxiety related vertigo (VSS-A) on total DHI scores. Using both VSS scores, the combination of vestibular and anxiety were highly correlated to dizziness handicap (r^2^ = 0.560, p<0.001). Interestingly, using a stepwise regression analysis, we find that the exclusion of VSS-A did not significantly affect the correlation (r^2^ = 0.557, p<0.001). This analysis strongly suggests that we cannot explain the DHI scores with a measure of anxiety alone raising the question of what other factors may be contributing to the dizziness.

### 4.2 Role of Vestibular Impairment in Anxiety

Another possible contributor to the symptoms of dizziness, loss of balance, poor coordination and sensitivity to noise could be underlying vestibular dysfunction in veterans with PTSD. Such symptoms are often attributed to the PTSD or anxiety and no attempt is made to examine a possible vestibular role. We recognize that these symptoms do not point to a specific pathologic etiology. We theorize a vestibular role based on prior work establishing an association between vestibular dysfunction and symptoms of dizziness, vertigo and disequilibrium [28–30] and findings that the vestibular ocular reflex in panic disorder patients is associated with increased gain and reduced time constant, which may be indicative of peripheral vestibular disease. [6] Our data supports a possible role for vestibular impairment in PTSD as well, since scores on the VSS-V were strongly correlated to both DHI and PCL.

In fact, examining the PTSD group, 81% would be classified as having likely vestibular dysfunction based on VSS-V scores [31] while only 19% of the non-PTSD group would be likely to have vestibular dysfunction, providing an odds ratio of 5.1 (N = 54, CI 1.4–18.468). These data suggests that impaired vestibular function, associated with vestibular symptoms, may be present in a subset of those with PTSD. However, this needs to be confirmed using direct measures of vestibular function in individuals with PTSD.

### 4.3 Interaction between Vestibular Dysfunction and Anxiety

Thirdly, there may be an interaction between vestibular dysfunction and anxiety which may result in the increased symptoms of dizziness. This relationship between the balance areas of the brain and anxiety has been studied. Neuroanatomical mapping by Balaban et al, Azzena et al, Liu et al has shown that connections exist between the vestibular nerve and areas of the brain associated with anxiety such as the hypothalamus, the cerebellum and the amygdala [2–5]. It is possible that this association may be mediated by avoidance, as this is a common symptom between patients with vestibular dysfunction or PTSD [32–34]. We performed a post hoc analysis to determine the extent to which avoidance symptoms are associated with dizziness and vertigo in PTSD. This analysis indicated that among the individuals with suggested vestibular impairment according to the VSS-V, 50% had avoidance versus only 13% in the group without vertigo, odds ratio of 7.0 (N = 42, CI 1.3–37.2).

With regard to the interaction between vestibular dysfunction and anxiety, two possible mechanisms that we could consider are 1) direct vestibular damage due to trauma; 2) secondary vestibular damage due to anxiety and stress.

#### 4.3.1 Direct vestibular damage due to trauma

Recent conflicts have been associated with exposure to physical explosive trauma from improvised explosive devices that can result in both head injury and PTSD [35–41]. Blast induced head injuries have been attributed to impairments of the vestibular system [35, 37, 41, 42]. When we examined the role of head injury within the PTSD and the non PTSD group, we found that the incidence of head injury was greater in the PTSD group versus the non PTSD group (72.5% versus 46.4%) respectively. These rates of head injury are similar to what has been previously established in the literature (55–67%), [42, 43]. The rates in the PTSD group are slightly higher in our cohort since this study focused on recruiting individuals with head injuries. To determine if the increased presence of TBI in the PTSD group was contributing to the increased vestibular symptoms in that group, we examined the effect of TBI on VSS-V and DHI scores, and found it had no significant effect. In addition, there was no interaction with PTSD status. These findings would suggest that TBI status was not a primary contributor to the increased rate of vestibular symptoms in the PTSD group.

It is surprising that Veterans with TBI did not report higher rates of symptoms associated with vestibular impairment, since previous literature has found increased incidence of vestibular impairment in this group [35, 37, 41, 42]. However there has only been one previous report using DHI scores in a Veteran with TBI. In this case Scherer et al reported that a blast exposed active duty corporal showed mild impairment on DHI within 40 days of a blast injury and his DHI score reduced from 14 to 2 after receiving vestibular rehabilitation exercises[38]. Thus, it remains unclear if VSS-V or DHI scores would be higher in Veterans with TBI that have been previously found to have vestibular impairment. One possible reason for this is due to natural adaptation to the impairment with time. Veterans in our study were reporting head injuries that had occurred years prior. Thus, it is possible that with adaptation and habituation of the vestibular system, symptom reports of vestibular impairment would decrease, and thus DHI and VSS-V scores would be lower. However, the lack of symptoms, possibly due to compensation, does not necessarily indicate that vestibular impairment is not present and points to the need to perform vestibular function tests.

#### 4.3.2 Secondary vestibular damage due to anxiety and stress

Another possible mechanism in the interaction between anxiety and vestibular function is the role of chronic stress on the vestibular system. Previous work has shown that injury to the vestibular apparatus has been associated with increased release of stress hormones such as vasopressin from the hypothalamic pituitary axis (HPA-axis) [44–46]. Activity within the HPA axis during chronic stress has been associated with mitigating adaptation within the vestibular system [45]. These data suggest that stress from the PTSD could impair vestibular function, thus resulting in increased VSS-V scores. However, without direct measures of vestibular function immediately post trauma and then after chronic stress exposure, it is difficult to know if chronic stress is underlying the development of vestibular symptoms.

In order to accurately understand the mediating factors that are contributing to the symptom reports of vestibular impairment, there is a need to perform direct measures of vestibular function. This will allow us to characterize if there is vestibular impairment to support the symptom reports.

## Study Limitations

The study findings are limited by the self-report nature of data collected. PTSD was identified based on subject report of symptoms within one month of study participation. While the PCL is widely used for capturing PTSD due to its psychometric strengths with regard to favorable sensitive/specificity ratio and test-retest reliability [10, 12, 13, 47], we are aware that the Clinician Administered PTSD Scale (CAPS) is considered the “gold standard” measure of PTSD. The CAPS was not used in this study as it is part of a larger program of research focused on post-deployment vestibular and autonomic dysregulation rather than PTSD specifically. Since this data was collected in addition to several additional tests, there was also a need to reduce subject burden by reducing the time for data collection. Additionally, with the exception of the neurobehavioral scale that was administered by a trained clinical researcher; the VSS-SF, DHI, and CMI were all self-reported data. We also recognize that the effect we observe with PTSD may also be apparent with other mental health disorders but this was not the focus of this work and we do not have sufficient data available to address that question. Similarly, since the head injury questionnaire was initiated, as data collection was ongoing, our exploration of the effect of head injury was limited. Further work is needed to determine whether the effect observed in our study is unique to PTSD, or may also be found in other mental health disorders.

## Conclusion

Symptoms of dizziness, vertigo and disequilibrium are a problem and a source of disability for veterans with PTSD. Previously, these symptoms in veterans with PTSD have been attributed to anxiety. However treatment for anxiety related dizziness has not been effective as treatment resistant dizziness persists in patients with anxiety [48]. It is possible that among veterans with PTSD and treatment resistant dizziness, underlying vestibular hypofunction may be a contributing factor. Our current sample set helped to identify these vestibular related symptoms among veterans with PTSD and that these symptoms are a major source of disability in this sample population.

In fact, examining the PTSD group, 81% would be classified as having likely vestibular dysfunction based on their VSS-V scores [31]. This data must be interpreted with caution since vestibular function tests were not performed. However, this is a substantial portion of our PTSD sample and this finding highlights a need to perform further investigation of the possible role of vestibular dysfunction in veterans with PTSD.

Data from this study also showed that anxiety contributes minimally to the symptoms of dizziness reported by veterans with PTSD. Performing a stepwise linear regression we found that only the vestibular subscale of the vertigo symptom scale was significantly correlated with the dizziness handicap inventory (r^2^ = 0.60 P<0.001). In fact the correlation between DHI and both subscales was r^2^ = 0.60. Removal of the anxiety subscale had no significant effect on the correlation, indicating that the anxiety subscale was not a major contributor to dizziness handicap.

Similarly, symptoms of dizziness could not be attributed to mTBI alone when co-morbid with PTSD. A chi square analysis of the frequency of endorsement of the symptom “feeling dizzy” in the last 30 days, indicated that a significant portion of the veterans with comorbid PTSD and mTBI endorsed dizziness. Only 11% of the complaints of dizziness could be explained by mTBI whereas PTSD explained 18.5% of the dizziness. The reported complaints of dizziness appear to be lower than what others have found, eg Chamelian et al identified 66.7% of patients with a TBI reported dizziness in a prospective study of TBI patients in a TBI clinic [49]. Similarly, in another prospective study, 81% of the patients with head injury reported symptoms of dizziness [50]. In both of these studies, the evaluation was performed shortly after the trauma, as early or as 72 hours after the event [50]. In other studies, lower rates of dizziness have been reported, ranging from 6% to 37% [38, 51, 52]. In the case of Scherer’s study, while 27% of the patients reported dizziness at any point since the injury, only 12% reported dizziness at the time of study participation. The findings of these studies in comparison with our study population highlight the point that the time frame of evaluation with respect to the head injury has an impact on the extent of symptoms reported. In our case, we asked the participants the extent to which they experienced dizziness within 30 days of study participation rather than since the injury occurred. In our study sample, the time frame since injury ranged from 5 to 12 years, with some participants who did not know the length of time since injury. It has been shown that recovery following a TBI can reduce the extent of symptoms in the years following the event [38, 53]. While we did not assess the extent of overall dizziness, since the traumatic injury, it is likely that this would have been much higher than their current symptoms, as shown by Griffiths et al [54]. For this particular study we were more interested in assessing current symptoms of the participants as we wanted to compare those with vestibular findings. Within our sample population, this finding highlights the non-specific nature of dizziness and a need for specific instruments to measure the sources of the dizziness when assessing these symptoms. This finding is also limited because it is based on one item on the NSI.

Overall this study highlights that direct measures of vestibular function are needed to determine if symptoms of dizziness in PTSD may be vestibular in origin. The findings of this study support the hypothesis that veterans with PTSD report more symptoms of vestibular impairment symptoms than veterans without PTSD. Poor vestibular function may also be a source of disability for veterans with PTSD. The relationship between PTSD and vestibular function may be mediated by components of anxiety, vestibular dysfunction and an interaction between the two. Further study of vestibular function in PTSD is required to identify underlying vestibular impairment in veterans with PTSD.

While it is of interest that there is increased symptom report among the veterans with PTSD, a correlation of PCL score and dizziness handicap score indicated that there is a significant relationship between the two items. With an increase in PTSD symptoms, there was also an increase in dizziness handicap symptoms indicating that veterans with PTSD have a reduced quality of life as a result of symptoms of dizziness. Since dizziness tends to be a non-specific symptom we also quantified the extent to which veterans experienced bouts of vertigo; as vertigo has been shown to be a key symptom among individuals with vestibular impairments [2, 28]. There was a strong correlation between PCL score and vertigo symptom score particularly on items relating to vertigo specifically. Further support for a vestibular role is also provided by the finding that quick head movements increased symptoms of dizziness to a greater extent in veterans with PTSD. These findings further strengthen the claim that vestibular impairment may be a significant contributor to the symptoms of dizziness or disequilibrium that was observed in our clinic.

Symptoms of avoidance are common among vestibular patients since their symptoms of dizziness can be exacerbated by various environments particularly theatres, shopping malls and supermarkets. We compared the extent of avoidance in veterans with and without PTSD when they were alone or accompanied in these environments. The results show that veterans with PTSD experience a greater level of discomfort and therefore avoid theatres, shopping malls and supermarkets. Additionally, avoidance was a risk factor for vertigo among the veterans with PTSD.

The results of this study indicate that veterans with PTSD report vestibular impairment symptoms and experience handicap as a result of these symptoms and in turn may avoid certain environments as a way to cope with these symptoms.

## Abbreviations

PTSD: Posttraumatic Stress Disorder
PCL: Posttraumatic Disorder Checklist
VSS-SF: Vertigo Symptom Scale- Short Form
CMI: Chambless Mobility Inventory
DHI: Dizziness Handicap Inventory
DSM-IV: Diagnostic Statistical Manual Version IV
VA: Veterans Affairs
NSI: Neurobehavioral Symptom Inventory
mTBI: mild Traumatic Brain Injury
CAPS: Clinician Administered PTSD Scale
